# Diagnosis of Von Hippel-Lindau Syndrome Via Pancreatic Cyst Fluid Next-Generation Sequencing

**DOI:** 10.14309/crj.0000000000001990

**Published:** 2026-02-17

**Authors:** Syedreza Haider, Jonathan Xia

**Affiliations:** 1 Division of Gastroenterology and Hepatology, University of Michigan, Ann Arbor, MI

**Keywords:** endoscopic ultrasound, pancreas cyst fluid analysis, pancreas cyst, Von Hipple-Lindau syndrome, pancreaSeq

## Abstract

Von Hippel-Lindau (VHL) syndrome is a rare autosomal-dominant tumor predisposition syndrome characterized by benign and malignant neoplasms across multiple organ systems. We report a case where next-generation sequencing (NGS) of pancreatic cyst fluid resulted in the first diagnostic clue to VHL. A 44-year-old woman presented with subarachnoid hemorrhage from cervical hemangioblastoma and incidental finding of multiple pancreas cysts. Endoscopic ultrasound with fine-needle aspiration was performed and standard cyst fluid analysis was nondiagnostic, but cyst fluid NGS identified a pathogenic VHL mutation. This case highlights the utility of cyst fluid NGS in uncovering hereditary cancer syndromes when conventional analyses are inconclusive.

## INTRODUCTION

Von Hippel-Lindau (VHL) syndrome is an autosomal-dominant tumor predisposition syndrome caused by pathogenic variants in the VHL gene on chromosome 3p25-26.^1^ It affects approximately 1 in 36,000 live births^2^ and is characterized by the development of benign and malignant neoplasms across multiple organ systems, including central nervous system hemangioblastomas, clear-cell renal cell carcinoma, pheochromocytomas, pancreatic cysts, pancreatic neuroendocrine tumors (pNETs), endolymphatic sac tumors, and retinal angiomas.^3,4^

The diagnosis of VHL is typically established through identification of a germline pathogenic variant in VHL in an individual with suggestive clinical features or a family history meeting established clinical criteria.^5,6^ However, VHL exhibits phenotypic heterogeneity and can occur de novo in up to 20% of cases.^1,7^ Moreover, somatic mosaicism, where the mutation is not detectable in peripheral blood, can complicate genetic diagnosis.^4^

Pancreatic involvement in VHL is common, seen in up to 70% of patients, and can present as cysts, serous cystadenomas, and pNETs.^7,8^ Although imaging and cyst fluid analysis with carcinoembryonic antigen (CEA), amylase, glucose, and cytology can aid in characterization, these conventional methods may be nondiagnostic in syndromic contexts.^7,8^ Molecular analysis of cyst fluid via next-generation sequencing (NGS) can identify driver mutations and, in rare instances, uncover underlying hereditary cancer.^9-11^

We present a case where NGS of pancreatic cyst fluid identified a pathogenic VHL mutation in a patient without prior diagnosis, leading to germline confirmation of VHL. This case underscores the role of cyst-fluid molecular testing not only in risk stratification for neoplasia but also as a tool for identifying hereditary syndromes, particularly when conventional diagnostics are unrevealing.

## CASE REPORT

A 44-year-old woman with no significant medical history and no known family history of cancer developed an abrupt, severe headache while traveling abroad. Neuroimaging demonstrated subarachnoid and intraventricular hemorrhage arising from a cervical junction hemangioblastoma. She underwent emergent external ventricular drainage and endovascular embolization, followed by suboccipital craniectomy and cervical laminectomy with tumor resection. She was subsequently repatriated to the United States for continued care and rehabilitation.

**Figure 1.**
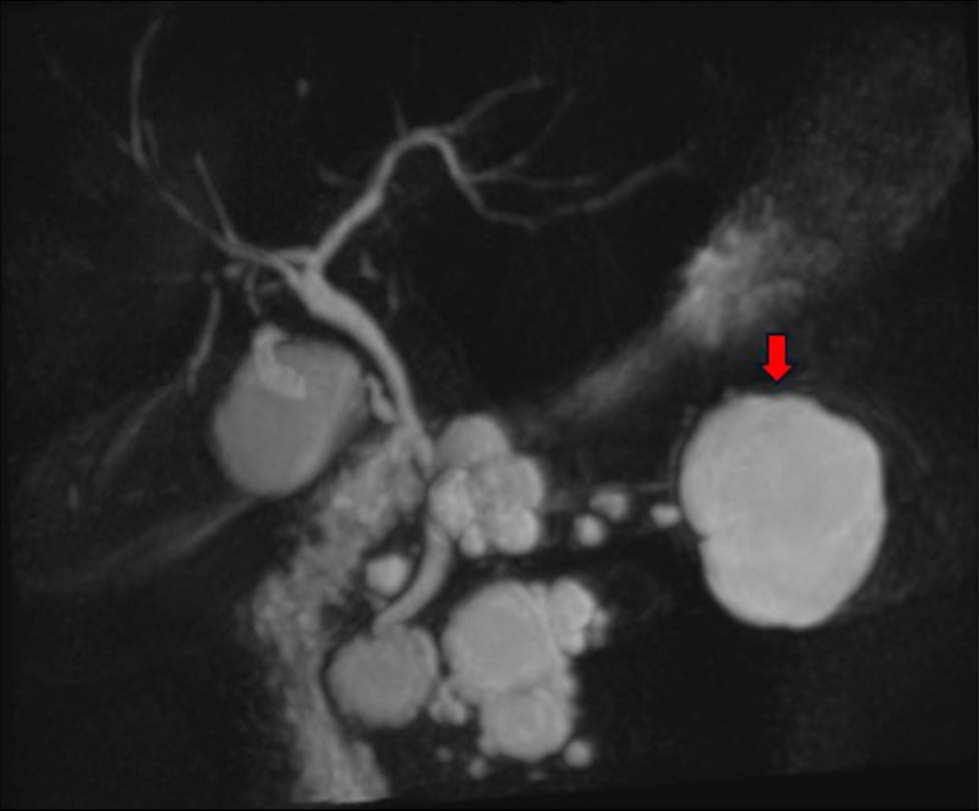
MRI/MRCP demonstrating multiple pancreas cysts. The arrow indicates the largest cyst in the distal pancreatic body that was targeted for biopsy in endoscopic ultrasound.

During her U.S. hospitalization, contrast-enhanced abdominal CT was obtained as part of a broader systemic evaluation after her initial rare presentation with a cervical hemangioblastoma. This revealed incidental findings of multiple pancreatic cysts and a complex left renal mass. Given the CT findings, a dedicated magnetic resonance cholangiopancreatography was performed that demonstrated many cystic lesions throughout the pancreas without mural nodules, enhancing soft tissue, or pancreatic ductal dilation (Figure 1). The largest cyst measured 4.8 cm in the pancreatic body; no solid pancreatic mass was identified. A left lower-pole cystic and solid renal mass measuring 4.4 cm × 2.9 cm showed posterior-inferior enhancing soft tissue without venous invasion or hydronephrosis. The radiology impression favored side-branch intraductal papillary mucinous neoplasms with short-interval MRI/endoscopic ultrasound (EUS) follow-up recommended, and a renal lesion concerning for neoplasm.

**Figure 2.**
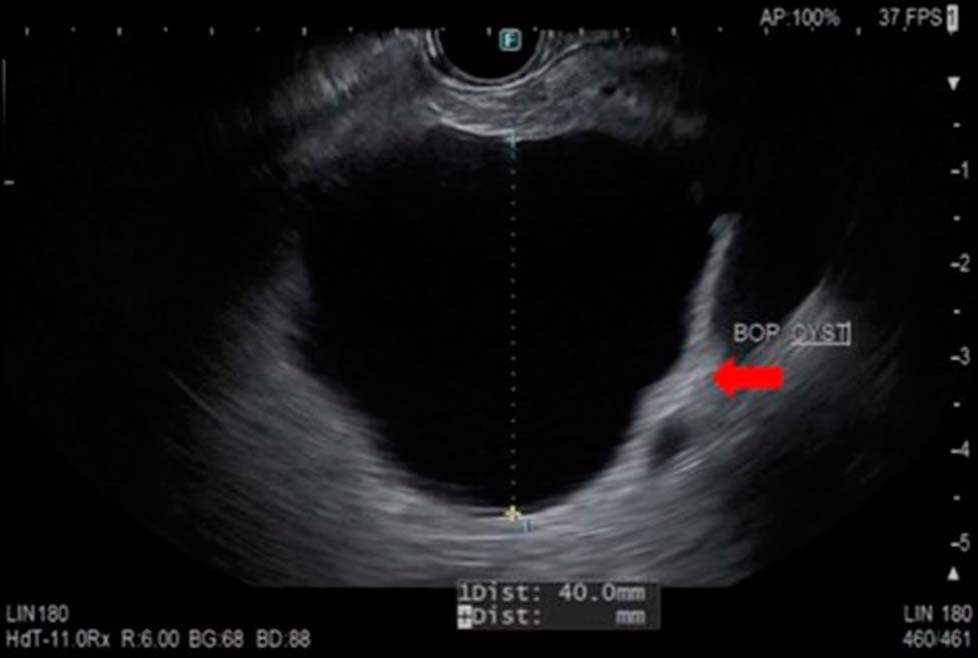
Endoscopic ultrasound of the largest cyst in the distal pancreatic body. The cyst was nonseptated and anechoic with subtle ductal communication. No high-risk features such as solid component or mural nodule were seen.

She had outpatient gastroenterology clinic evaluation of her pancreas cysts followed by EUS, which confirmed multiple pancreas cysts in the head, uncinate, body, and tail of the pancreas. Endosonographic exam of the largest cyst seen on MRI in the body of the pancreas showed that it was a unilocular, anechoic cyst without septations, solid components, or associated pancreas duct dilation (Figure 2). Overall endosonographic impression of this cyst was consistent with an intraductal papillary mucinous neoplasm without high-risk features other than its size. The pancreatic parenchyma was otherwise normal without calcifications or masses.

**Figure 3.**
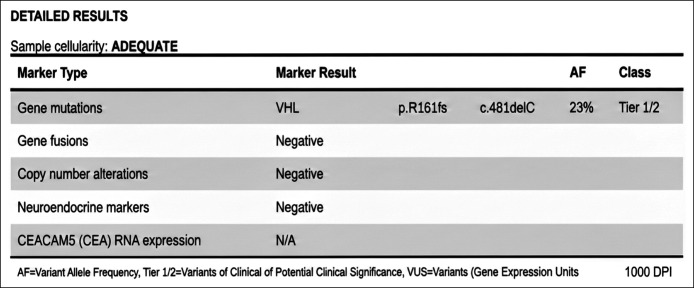
Cyst-fluid PancreaSeq results. NGS of pancreas cyst fluid identified a pathogenic, protein-truncating VHL variant (c.481delC, p.R161fs; AF 23%). NGS, next-generation sequencing; VHL, Von Hippel-Lindau.

Fine-needle aspiration of the largest cyst using a 22-gauge needle and 15 milliliters of thin, clear fluid was aspirated and sent for cytology, amylase, CEA, glucose, and cyst-fluid NGS (PancreaSeq). NGS of cyst fluid was pursued despite the benign appearance of the cyst due to her atypical complex presentation. As expected, cytology revealed no atypical or malignant cells, with macrophages consistent with cyst contents. Fluid chemistries were amylase 46 IU/L, CEA <1 ng/mL, and glucose 99 mg/dL; a profile inconsistent with mucinous cyst. PancreaSeq identified a pathogenic, protein-truncating VHL variant (c.481delC, p.R161fs; AF 23%), with no KRAS, GNAS, BRAF, or RNF43 alterations (Figure 3). In the context of her cervical hemangioblastoma and complex renal mass, this result prompted expedited referral to medical genetics. Subsequent germline testing confirmed a diagnosis of Von Hippel-Lindau disease.

At the time of reporting, the patient is engaged in neurorehabilitation and is participating in genetic counseling to initiate a VHL surveillance plan (retinal, central nervous system, abdominal, and endocrine screening per guideline). She is currently discussing with her oncologist on initiating belzutifan, a selective hypoxia-inducible factor-2α inhibitor that targets the downstream hypoxia pathway activated in VHL-associated tumors.^4^

## DISCUSSION

This case underscores the diagnostic impact of cyst-fluid molecular analysis in identifying hereditary cancer syndromes when standard tests are inconclusive. In this patient, both initial imaging and endosonographic assessment pointed to a low-risk benign cyst. These findings would typically prompt conservative management and routine imaging surveillance rather than further investigation.^8,12^ Although there were suspicions of a broader systemic disease during her initial hospitalization, at the time of her GI evaluation and EUS almost 6 months posthospitalization, she still has not been referred for genetic counseling. Thus, the application of NGS to the cyst fluid proved pivotal, rapidly identified a pathogenic VHL variant, which in this clinical context immediately shifted the diagnostic focus and led to urgent referral to medical genetics and germline confirmation of VHL syndrome.^9,10^

Pancreatic involvement in VHL is common, but it often presents as serous cystadenoma or neuroendocrine tumors on imaging.^13^ This makes early diagnosis challenging especially when family history is negative and extrapancreatic manifestations have not yet been recognized.^7^ In the present case, the pancreas lesions did not have endosonographic findings typical of either serous cystadenoma or pancreas neuroendocrine tumor and appeared like a low-risk nonseptated IPMN. VHL loss-of-function variants in cyst fluid are strongly associated with pancreatic serous cystadenoma and are uncommon in mucinous cysts, making VHL alterations in cyst fluid highly informative for cyst-type classification. Importantly, in the setting of concurrent VHL-associated manifestations, identification of a pathogenic VHL variant in cyst fluid served not only as a cyst-type discriminator but also as a syndromic clue prompting expedited germline evaluation. Thus, the same molecular signal that often supports a benign serous cyst diagnosis can, when interpreted in the appropriate clinical context, facilitate timely recognition of underlying VHL disease and initiation of syndrome-directed surveillance^10,11.^

In our practice, cyst-fluid NGS is not routinely performed for all pancreatic cysts. Rather, it is selectively applied when standard fluid chemistries, cytology, and imaging are inconclusive, when cyst has high-risk features but EUS findings are equivocal, or when there is concern for an underlying hereditary syndrome. Current literature supports the use of NGS in these settings because it improves diagnostic accuracy and risk stratification, helps distinguish mucinous from nonmucinous cysts, and identifies molecular alterations associated with early neoplasia or syndromic disease.^9,10,14–16^

This case highlights 2 important clinical lessons. First, molecular analysis of pancreatic cyst fluid extends beyond risk stratification for neoplasia. It can serve as a diagnostic tool for a subset of hereditary syndromes, especially when clinical and radiologic findings are ambiguous or accompanied by unexplained vascular lesions.^4,6^ Second, for patients with indolent cysts but complex or unexplained medical histories, the threshold for advanced molecular diagnostic testing should be low, as the consequences of a missed hereditary syndrome are significant. As molecular and next-generation sequencing based diagnostic platforms become increasingly accessible, their integration into the workup of pancreatic cystic lesions may facilitate timelier and more precise personalized care. Clinicians should recognize the value added by cyst-fluid NGS when conventional evaluation fails to account for the clinical complexity, as illustrated by the present case.

## DISCLOSURES

Author contributions: All authors meet ICMJE criteria (substantial contributions; drafting/revision; final approval; full accountability). S. Haider: conceptualization; data curation; chart review; drafting of manuscript; figure/table preparation; revision for important intellectual content. J. Xia: Endoscopic procedure and image acquisition; interpretation of endoscopic, cytologic, and molecular results; supervision; critical revision for important intellectual content. J. Xia is the article guarantor, accept full responsibility for the integrity of the work, had access to the data, and controlled the decision to publish.

Previous presentation: This case was presented as an abstract at the *American College of Gastroenterology* (ACG) Annual Scientific Meeting (Pheonix, AZ); abstract to be published in *The American Journal of Gastroenterology* supplement.

Financial disclosure: None to report.

Informed consent was obtained for this case report.

## ABBREVIATIONS:

CEA: carcinoembryonic antigen
CT: Computed Tomography
EUS: endoscopic ultrasound
IPMNs: Intraductal papillary mucinous neoplasms
MRCP: Magnetic resonance cholangiopancreatography
MRI: Magnetic Resonance Imaging
NGS: next-generation sequencing
pNETs: pancreatic neuroendocrine tumors
VHL: Von Hippel-Lindau
